# Research on regeneration methods of animal glue waste sand for foundry

**DOI:** 10.1098/rsos.172270

**Published:** 2018-05-16

**Authors:** Li Ying-min, Wang Tian-shu, Liu Wei-hua

**Affiliations:** School of Materials Science and Engineering, Shenyang University of Technology, Shenyang, 110870, People's Republic of China

**Keywords:** animal glue waste sand, waste sand regeneration, loss on ignition, clay content, reclaimed sand

## Abstract

Sand moulds are used in the casting process. However, after heating, the binder in the sand loses the binding properties, and the most part of the foundry sand has to be discarded from the process. The waste foundry sand after the regeneration can be recycled, and reclamation can reduce the production cost and lower waste emissions. The objective of this work was to investigate the possibility of reusing the animal glue binder waste foundry sand from the study of three regeneration methods. Studies with the waste foundry sand and reclaimed sand were performed in order to compare the results obtained with raw sand. The characterization of the samples was performed by scanning electron microscopy and energy dispersive spectroscopy. Results show that the waste sand was regenerated by mechanical regeneration, thermal regeneration and wet reclamation, respectively. The reclaimed sands have a better performance than the waste foundry sand, and are similar to raw sand. Further, the wet regeneration method is the best one among the three methods.

## 1. Introduction

Foundry sands are commonly used in casting processes by metal foundries to form moulds in which molten metal is poured. After cooling, the sand moulds are broken, and the finished iron products are removed. Binders are added to the sands to maintain the shape of the mould during pouring and cooling.

In the foundry industry in China, these processes create a yearly average of 40 million tons of castings. However, 1 ton of castings can produce 1.2–1.4 tons of solid waste, 40–60% of which is made up of core and moulding sands. Reclamation is becoming a necessity in foundry operations [1–3]. There are three methods for the recovery of waste foundry sands for core operations: dry mechanical reclamation, thermal reclamation and wet reclamation [4–7].

In this study, we used an organic binder, namely animal glue binder, which is a non-toxic, biodegradable natural polymer material binder [8]. In this paper, mechanical reclamation, thermal reclamation and wet reclamation were used to regenerate animal glue waste foundry sand, respectively. The sample was characterized and analysed by scanning electron microscopy (SEM) and energy dispersive spectroscopy (EDS). Through laboratory analysis, we characterized the physical and chemical aspects of the reclaimed sand for comparison with raw sand and waste foundry sand. By this, we can also choose the best regeneration method among the three methods.

## 2. Material and methods

**Figure 1.**
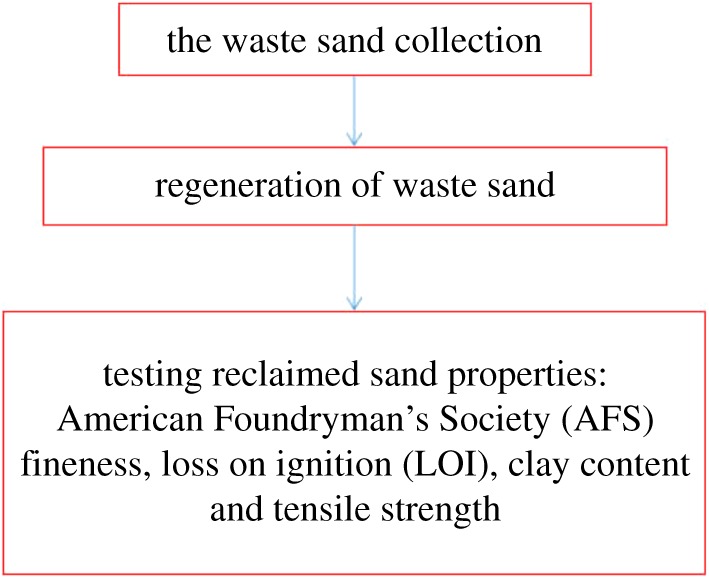
The regeneration process for checking sand reusability.

The regeneration process that was followed for checking sand reusability is given in figure 1.

The raw sand came from Dalin in China. The animal glue waste sand came from laboratory simulation pouring; we used the mechanical regeneration, thermal regeneration and wet regeneration methods, respectively. Each regeneration was carried out with 2000 kg of waste sand and repeated three times. In the next step we obtained the reclaimed sand, which was followed by testing of its properties for AFS fineness, loss on ignition (LOI), clay content and tensile strength.

LOI is the measurement of the weight change of a sample, according to the national testing standard of moulding sand performance [9]. A sample of 10 ± 0.001 g of reclaimed sand from each reclamation method was taken and heated for 1 h in an oven at 1000°C to test LOI. The LOI value is obtained based on the calculation in the following equation. The final LOI value was the average of the three samples.

2.1 $$\text{LOI}=\frac{m_{1}-m_{2}}{m}\times 100\%,$$

where *m* is the starting weight of material (g); *m*_1_ is the starting weight of the crucible with material (g); *m*_2_ is the fired weight of the crucible with material after firing and subsequent cooling (g).

The clay content of sand was determined by the methylene absorption method. This test consists of titrating the moulding sand (5 g mixed in 100 ml of water and 1.5 ml of sulphuric acid) with methylene blue, as published by the national testing standard of moulding sand performance. Moreover, the final clay content value was the average of the three samples.

American Foundryman's Society (AFS) fineness was determined by sieving, with reference to a grading test in which the weight proportions are separated by passing the dried sample (100 g) through a set of standard testing sieves (GB-T2684-2009).

The tensile strength of the prepared ‘8' samples was measured by an SWY testing machine. Firstly, a sample was placed on the testing machine. Then, the sample was gradually loaded until it was broken and its tensile strength value could be recorded in the instrument. The final tensile strength was the average of the five samples.

## 3. Characteristics of animal glue waste sand

According to the national testing standard of moulding sand performance, the properties of waste foundry sand including clay content, AFS fineness, LOI and tensile strength were tested after the waste sand had been screened and had undergone magnetic separation. The test results are presented in table 1.

**Table 1.** Test results of raw sand and waste animal glue sand.

sand type	LOI (%)	clay content (%)	AFS fineness	tensile strength (MPa)
raw sand	0.38	0.26	50	2.67
waste sand	0.70	1.20	52	0.85

**Figure 2.**
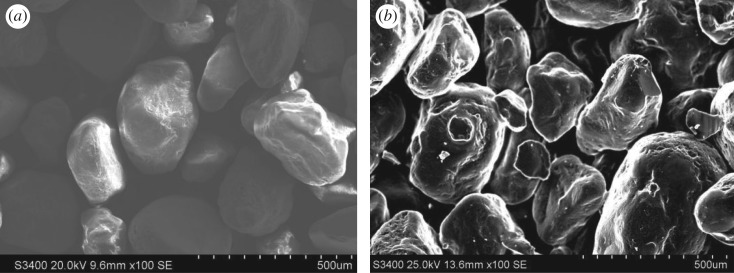
Surface morphology of sand at SEM observation: (*a*) raw sand; (*b*) waste sand.

As given in table 1, the LOI and clay content of the waste sand were much higher than those of the raw sand. If the waste sand is used again, the air permeability of the moulding sand becomes poor, and the surface of the casting has a hole. Therefore, the waste foundry sand needs regeneration. The surface morphologies of the waste sand and raw sand are shown in figure 2.

It can be seen from figure 2 that the surface of the animal glue waste sand is coated with a large number of residual binder attachments and its surface is rough. Furthermore, the residual binder attachments form films on the surface of waste sand, so the waste sand surface energy decreases. The thicker the inert film on the surface of the waste sand, the smaller is the surface and the lower is the activity of the sand.

## 4. Mechanical regeneration and mechanical reclaimed sand characteristics

Mechanical regeneration uses mechanical force to remove the binder film on the surface of waste sand. Mechanical regeneration was performed with the roller-type sand mixer. This regeneration is suitable for the waste sand with a brittle binder film. During the moulding of animal glue sand, a large number of water molecules evaporate from the sand by heating, which makes the binder film change from toughness to brittleness. The residual brittle film on the waste sand surface can easily fall off through the collision and friction between sand grains, and sand and equipment, so as to realize the regeneration. The mechanical regeneration time measurements were carried out and the test results are given in table 2.

**Table 2.** Performance of reclaimed sand after mechanical reclamation.

mechanical regeneration time (min)	LOI (%)	clay content (%)	AFS fineness
10	0.75	0.78	48
15	0.69	0.68	49
20	0.54	0.54	50
25	0.42	0.45	50
30	0.40	0.43	51
35	0.39	0.40	52
40	0.38	0.37	52

As given in table 2, the clay content and LOI of the waste sand are reduced after mechanical regeneration, but it takes a long time. The binder film of the waste sand surface was removed by the roller compaction machine; however, with increase of the rolling time, the reclaimed sand size became thinner and AFS fineness increased. The mechanical regeneration time was 25 minutes.

**Figure 3.**
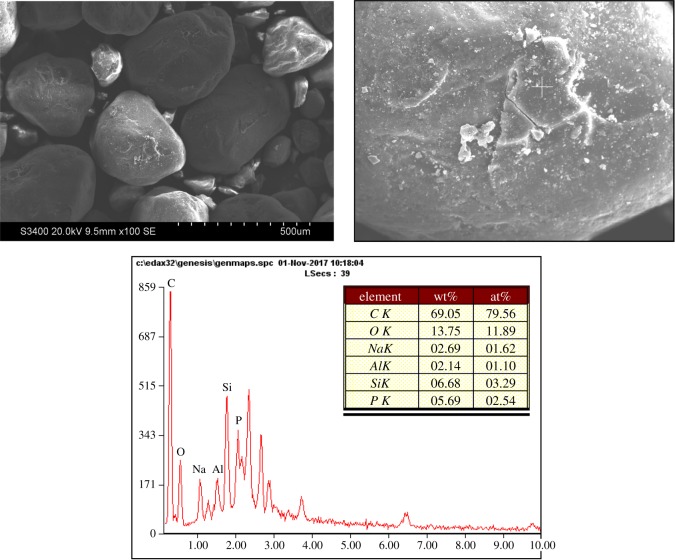
The SEM and EDS of mechanical regeneration reclaimed sand.

The surface morphology and element content of the reclaimed sand by mechanical regeneration are shown in figure 3. It can be seen that the mechanical regeneration reclaimed sand surface has become smoother and cleaner than the waste sand, and there is a little of the residual binder around the reclaimed sand.

## 5. Thermal regeneration and thermal reclaimed sand characteristics

Thermal regeneration is a method which is used to break up or burn the residual binder on the surface of the waste sand by heating to a certain temperature in a baking furnace, and this is suitable for a flammable organic binder. Animal glue binder is a natural macromolecule compound, which can be regenerated by thermal means to make the residual binder film decompose and be removed, so as to achieve the purpose of regeneration. Thermal regeneration is carried out in a muffle furnace.

The influence of the temperature on the reclaimed sand was studied, and the results are presented in table 3.

**Table 3.** Performance of reclaimed sand after different reclamation temperatures.

temperature (°C)	LOI (%)	clay content (%)	AFS fineness
300	0.45	0.55	51
400	0.39	0.43	50
500	0.36	0.40	50
600	0.33	0.37	52
700	0.30	0.34	52
800	0.27	0.31	52

Temperature is the most important factor that affects thermal reclamation. If the temperature is too low, the animal glue binder on the surface of the sand would not decompose. If the temperature is too high, it would increase the thermal reclamation cost.

As presented in the table, the clay content and LOI in the reclaimed sand both decreased when the reclamation temperature was increased. The main reason for this is that the decomposition rate of the residual binder on the waste sand surface increased as the temperature was raised. So 400°C was selected as the thermal regeneration temperature.

The influence of the reclamation time on the reclaimed sand was also tested, and the results are given in table 4. From this table, after 15 min thermal reclamation, the LOI of the reclaimed sand was similar to that of raw sand, and the clay content was lower than that of the other types of reclamation.

**Table 4.** Performance of reclaimed sand after different reclamation times.

time (min)	LOI (%)	clay content (%)	AFS fineness
5	0.53	0.65	49
10	0.45	0.43	50
15	0.36	0.31	50
20	0.35	0.37	50
25	0.34	0.42	52
30	0.33	0.54	52

**Figure 4.**
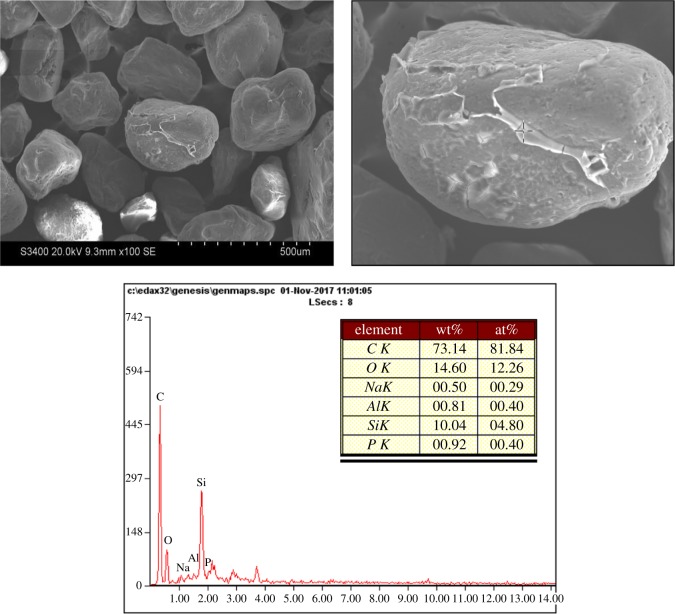
The SEM and EDS of thermal reclamation reclaimed sand.

From the surface of the thermally regenerated sand (figure 4), it can be seen that the thermal regeneration reclaimed sand surface became cleaner than that of mechanical regeneration reclaimed sand, and there is a little residual film around the reclaimed sand.

## 6. Wet regeneration and wet reclaimed sand characteristics

**Figure 5.**
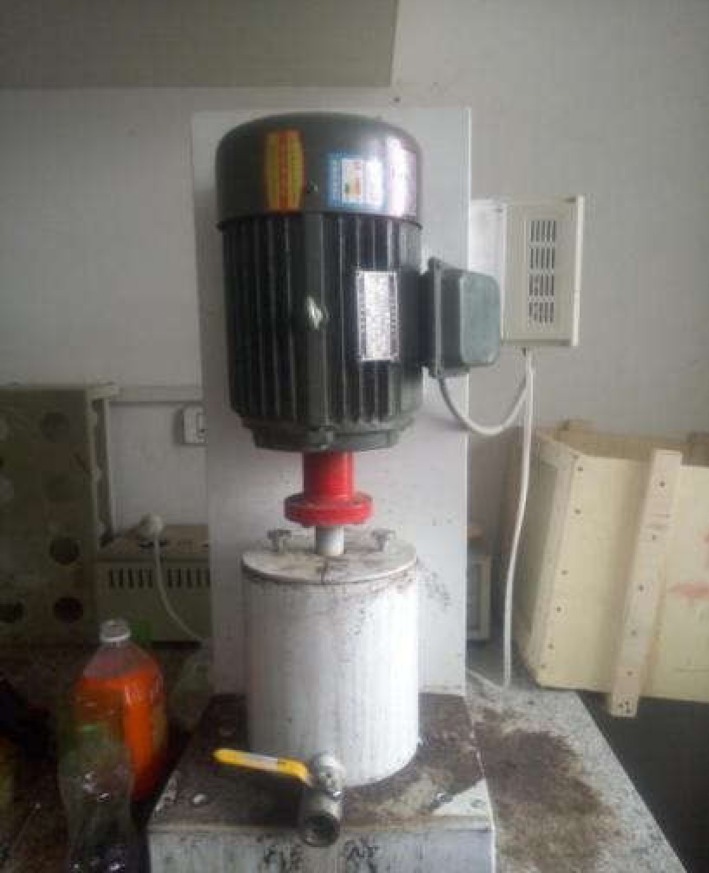
The swaging machine.

Under mechanical agitation and water scrubbing, the bond film on the waste sand can be removed and dissolved so as to achieve the effect of reclamation. Wet regeneration was carried out with a swaging machine which was made in the laboratory (as shown in figure 5).

The clay content, LOI and AFS fineness of the sands were tested by their different sand–water ratios and the results are given in table 5.

**Table 5.** Effects of the sand–water ratio on reclaimed sand characteristics.

sand–water ratio	LOI (%)	clay content (%)	AFS fineness
2 : 1	0.44	0.45	50
2 : 2	0.39	0.35	50
2 : 3	0.36	0.33	50
2 : 4	0.35	0.30	50
2 : 5	0.34	0.27	50
2 : 6	0.33	0.24	50

When the water flow increased, the rotational friction of water to the waste sand is more intense and can effectively remove or dissolve the bond film on the surface of the waste sand. The activity of the surface of the sand can be increased. As illustrated in table 5, the clay content and LOI of the reclaimed sand decreased as the sand–water ratio were increased, and after 2 : 3, the clay content and LOI changed less.

The influence of the wet reclamation time on the reclaimed sand was tested, and the results are presented in table 6. With the increase in regeneration time, the LOI and clay content were decreased. In addition, as the time increases, the faster will the bond film be removed or dissolved in the water, and the LOI and the clay content were more similar to those of the raw sand. After 11 min, the clay content and LOI changed less.

**Table 6.** Effects of the wet regeneration time on reclaimed sand characteristics.

time (min)	LOI (%)	clay content (%)	AFS fineness
2	0.56	0.78	51
5	0.47	0.58	50
8	0.40	0.49	50
11	0.37	0.33	50
14	0.35	0.30	51
17	0.33	0.30	53

Meanwhile, the influence of stirring speed on the reclaimed sand also was tested. Table 7 shows that the LOI and clay content of the reclaimed sand will decrease remarkably if the waste foundry sand is processed by 280 r.p.m. With a faster stirring speed, the waste sand collision was greater, and the residual binder film of the sand surface was removed faster. So the wet regeneration effect was obviously increased.

**Table 7:** Effects of the stirring speed on reclaimed sand characteristics.

stirring speed (r.p.m.)	LOI (%)	clay content (%)	AFS fineness
80	0.55	0.54	47
120	0.47	0.49	49
160	0.44	0.44	49
200	0.40	0.38	50
240	0.38	0.34	50
280	0.35	0.29	50
320	0.33	0.27	51
360	0.33	0.27	51
400	0.33	0.26	52

**Figure 6.**
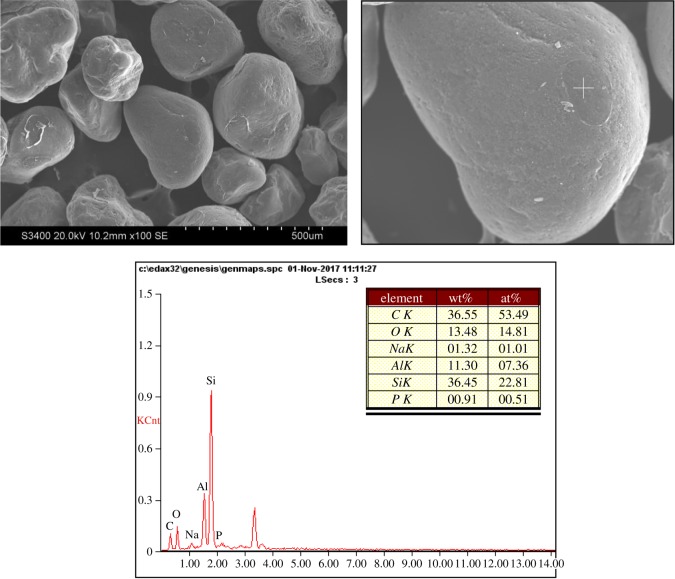
The SEM and EDS of wet reclamation reclaimed sand.

The SEM and EDS of reclaimed sand by wet reclamation are shown in figure 6. It can be seen that the animal glue binder film was removed from the surface, and there was hardly any residual binder around the reclaimed sand. Wet reclamation reclaimed sand surface was smooth and clean.

## 7 Comparison of three regeneration methods

In table 8, properties of reclaimed sand tested after the three regeneration methods are reported. They are as follows: (1^#^) 25 min of mechanical regeneration; (2^#^) 400°C and 15 min of thermal regeneration; (3^#^) 2 : 3 sand–water ratio, 280 r.p.m. and 11 min of wet regeneration.

**Table 8:** Performance of the reclaimed sand.

sand type	LOI (%)	clay content (%)	AFS fineness	tensile strength (MPa)
raw sand	0.38	0.26	50	2.63
waste sand	0.70	1.20	48	0.55
1^#^	0.42	0.45	50	2.00
2^#^	0.36	0.31	50	2.41
3^#^	0.35	0.29	50	2.60

As presented in the table, the performance of three reclaimed sands was similar to that of raw sand. The sequence of regeneration methods with regard to effect on the LOI and the clay content was wet reclamation, thermal regeneration and thermal regeneration. The LOI (0.35%) and the clay content (0.29%) of wet reclaimed sand were the lowest. Moreover, mechanical regeneration and thermal regeneration have a long-time, high energy consumption and low regeneration efficiency. The wet regeneration has a short-time, high regeneration efficiency and simple equipment. The waste water can be recycled after simple treatment. Therefore, wet regeneration may be more suitable for large-scale production.

## 8. Conclusion

In this study, we characterized waste foundry sand and raw sand. We also found that the three regeneration systems affect the nature of the reclaimed sand differently, most notably the LOI and the clay content.

Our obtained results demonstrate that waste sand can be regenerated by mechanical regeneration, thermal regeneration and wet regeneration. Moreover, the sequence of regeneration methods with regard to the effect on the LOI and the clay content was wet reclamation, thermal regeneration and thermal regeneration. All reclaimed sands were similar to the raw sand. However, the wet regeneration method is the best one among the three methods and it may be more suitable for large-scale production.

## Data accessibility

This article does not contain any additional data.
